# Care for patients with severe mental illness: the general practitioner's role perspective

**DOI:** 10.1186/1471-2296-10-29

**Published:** 2009-05-06

**Authors:** Marian JT Oud, Jan Schuling, Cees J Slooff, Klaas H Groenier, Janny H Dekker, Betty Meyboom-de Jong

**Affiliations:** 1 Department of General Practice, University Medical Centre Groningen, Groningen, The Netherlands; 2 Department of Psychiatry, University Medical Centre Groningen, Groningen, The Netherlands; 3 Mental Health Centre Drenthe, Assen, The Netherlands

## Abstract

**Background:**

Patients with severe mental illness (SMI) experience distress and disabilities in several aspects of life, and they have a higher risk of somatic co-morbidity. Both patients and their family members need the support of an easily accessible primary care system. The willingness of general practitioners and the impeding factors for them to participate in providing care for patients with severe mental illness in the acute and the chronic or residual phase were explored.

**Methods:**

A questionnaire survey of a sample of Dutch general practitioners spread over the Netherlands was carried out. This comprised 20 questions on the GP's 'Opinion and Task Perspective', 19 questions on 'Treatment and Experiences', and 27 questions on 'Characteristics of the General Practitioner and the Practice Organisation'.

**Results:**

186 general practitioners distributed over urban areas (49%), urbanised rural areas (38%) and rural areas (15%) of the Netherlands participated. The findings were as follows: GPs currently considered themselves as the first contact in the acute psychotic phase. In the chronic or residual phase GPs saw their core task as to diagnose and treat somatic co-morbidity. A majority would be willing to monitor the general health of these patients as well. It appeared that GP trainers and GPs with a smaller practice setting made follow-up appointments and were willing to monitor the self-care of patients with SMI more often than GPs with larger practices.

GPs also saw their role as giving support and information to the patient's family.

However, they felt a need for recognition of their competencies when working with mental health care specialists.

**Conclusion:**

GPs were willing to participate in providing care for patients with SMI. They considered themselves responsible for psychotic emergency cases, for monitoring physical health in the chronic phase, and for supporting the relatives of psychotic patients.

## Background

Severe mental illnesses (SMI), especially the schizophrenia spectrum and affective psychotic disorders present themselves in a wide variety of clinical signs and symptoms. The common denominator of these disorders is the loss of contact with reality and people of importance to them. Therefore these patients demand specific attention for their wellbeing and health problems.

For treatment, a crucial distinction between the acute phase and the chronic phase must be made. During the acute phase, when positive symptoms and reality distortion prevail and a psychotic crisis arises, GPs are often the first to offer medical assistance. Their role is to recognize the signals of a developing psychosis and to start appropriate treatment immediately [1,2]. This usually results in a referral to an emergency psychiatric consultation and, less often, the prescription of an antipsychotic drug [3].

In the chronic phase, the role of the GP is less well-defined. Psychiatric treatment is more or less restricted to infrequent medication checks by the psychiatrist and to the support by a community psychiatric nurse with regard to self care, daily structure and activities and reintegration of the patient in the community.

Long term chronic psychotic disorders are often accompanied by a loss of cognitive abilities, such as disturbances in perception, retardation, and executive functions. Psychotic patients may not be able to recognise certain physical phenomena as a symptom of a co-morbid disease [4], and they often find it difficult to communicate their physical needs and problems and to arrange appointments for themselves [5-7]. As a consequence, they seek help at a relatively late stage. These facts underscore the importance that care provided by GPs should be easily accessible. Furthermore, the GP should be alert on somatic co-morbidity, and pay specific attention to the differential diagnosis combined with the patient's physical condition [8].

Not only patients with SMI, but also their carers experience a lot of stress. They visit their GPs with their concerns as well [9,10]. GPs are aware of this and they feel that providing support for families of patients is one of their core tasks [11]. Mental health education for family members appears to be an effective intervention in the treatment of psychosis [12,13].

Patients with a chronic psychosis often suffer from somatic co-morbidities [14-16], and have a higher death risk [17]. Disease-related factors including chronic stress, smoking, drug abuse, life-style habits and lack of exercise contribute to this as well [18,19]. The use of antipsychotic drugs can also result in overweight and diabetes mellitus [20-23].

GPs seem not to be aware of the high rate of physical illness in patients with severe mental illnesses, nor do they pay specific attention to monitoring and treating somatic co-morbidity in this group of patients [24]. Internationally, it is currently acknowledged by psychiatrists that general health-care needs in patients with severe mental illness are neglected. Also, that the integration of general somatic and psychiatric care services is less than optimal [25,26]. However, there is a lack of consensus as to which health care professionals should be responsible for the prevention and management of co-morbid somatic illnesses in SMI patients [27]. Psychiatrists think that the monitoring of metabolic disorders as a possible side-effect of antipsychotic drug is their responsibility [27-29], but not necessarily the medical treatment. The updated UK guideline for schizophrenia states that for people with schizophrenia, just as for other high-risk groups, regular physical checks and health advice are an essential primary care contribution to their treatment and management [30]. Adherence to the guideline on monitoring risk factors in patients taking second-generation antipsychotics, appears to be low [31].

Dutch GPs have no specific management policy in the guidance of SMI patients; their current multidisciplinary guidelines on schizophrenia do not give them a sufficient directive [32].

Qualitative research on opinions of experienced GPs identified the factors which influenced their attitudes to providing care for patients with psychotic disorders [11]. Patient behaviour factors like aggression and drug abuse were perceived as being as difficult to manage and sometimes threatening, while involvement with the patient's family was a stimulus for the GP to do as much as he could. Also a good collaboration with acute psychiatric services was indispensable for GPs to feel competent in managing a psychotic crisis.

In order to check current practice among GPs and the need for a set of guidelines, we posed the following questions:

1. What part of the health care should be provided by GPs for patients with severe mental illnesses, both in the acute and the chronic phase?

2. Do GPs consider themselves sufficiently equipped to provide this care, and if not, in which areas do they need more training?

3. How do GPs manage their care for patients with SMI in practice, and how close is their collaboration with mental health services?

## Methods

### The survey instrument

After reviewing the literature [32-40] a questionnaire was developed regarding the GP's role perspectives, his treatment of SMI patients, his personal details and his practice organisation.

The term 'Severe Mental Illness' was confined to patients with psychotic symptoms related to the schizophrenia and affective psychotic spectrum.

A division was made between 'Providing Emergency Care' and 'Providing Care in the Chronic Phase'. Questions about GP's attitude to periodic health checks were divided into three aspects: physical health, psychosocial well-being and psychiatric symptoms.

The draft questionnaire was reviewed by an expert panel and was tested in a pilot study by interviewing four experienced general practitioners. The purpose of this was to test for ambiguity, the relevance of the questions, and the difficulties in answering them. The revised questionnaire was divided into three sections: Section 1: 'Opinion and Task Perspective', Section 2: 'Treatment and Experience' and Section 3: 'Personal Details of the GP and Practice Organisation'.

An ordinal five-level Likert scale with variations between positive and negative statements was used for answering eleven questions on 'Opinion and Task Perspective', five of the questions on 'Treatment and Experiences', and six questions on 'Characteristics of the GP'. The other 43 questions were multiple choices, except for the questions on age of the respondent and an estimate of the number of registered patients who were susceptible to psychosis.

The secretary of the Medical Ethics Committee of the University Medical Centre Groningen stated upon being consulted that it was not necessary to obtain the consent of the committee.

The questionnaire was distributed in June 2007. It took 20–30 minutes to complete the questionnaire. Participation was voluntary and anonymous. All GP's received a reminder four weeks after the distribution.

### The sample

The research was conducted among GPs who provided continuous care, i.e. established GPs and GPs in service of established GPs. Locum GPs were excluded from the research. A random sample of 700 GPs was taken from the database of Netherlands Institute for Health Service Researche (NIVEL).

### Data analysis

The data were processed using the software programme SPSS 14.0; which was also used for the statistical analysis. The answers on the ordinal five-level Likert scale were translated into a three point scale, measuring either a positive or a negative response to a statement. The answers to the questions on 'Opinion and Task Perspective' and on 'Treatment and Experiences' were subjected to a factor analysis. The factors found were correlated with personal details and treatment aspects of GPs using multivariate analysis.

## Results

A total number of 186 completed questionnaires was returned (27%). Nine questionnaires were undeliverable. Of the respondents, 62% were male, the average work experience was 18 years (minimum 2 years, maximum 35 years) and the average age was 49 years. 22% worked single-handed, 32% worked in two-man practice, 29% in a group practice and 17% in a primary health care centre. These data are consistent with the national figures on Dutch GPs from NIVEL.

Of the respondents, 52% of GPs worked part-time. 40% were also GP trainers, and 31% had psychiatric work experience. 61% had easy access to a community psychiatric nurse, whose main task was to advise the patient on problem solving. They rarely supervised patients with severe mental illnesses (13%). The average number of patients in a GP practice who were thought to be susceptible to psychosis was about 20 (minimum 0, maximum 200). The mean size of a Dutch GP practice covers 2350 inhabitants. Concerning practice location: 49% was located in an urban area, 38% in an urbanised rural area, and 15% in a rural area.

### Opinion and Task Perspective (table 1)

**Table 1:** Opinion and Task Perspective (N = 186)

	Agree	Neutral	Disagree
**Tasks in the acute and long term phase**			

For acute confusion, the GP is the first contact	74%	19%	7%
I feel responsible for the care for chronic psychiatric patients in my practice	58%	23%	19%
I think it is my job to check on psychiatric patients' ability to take care of themselves	37%	30%	33%
I think I should monitor somatic co morbidity in chronic psychiatric patients	81%	13%	6%

**Care for family**			

I think it is my job to support the family of a chronic psychotic patient	74%	17%	9%
I think it is my job to provide information on the clinical picture to the family of a chronic psychotic patient	58%	26%	16%

**Self-experienced competencies**			

I feel competent in making contact with the patient in a psychotic crisis	46%	35%	19%
I feel competent in communicating with the family in a psychotic crisis	85%	11%	4%
I feel competent in intervening in a crisis situation	52%	33%	15%
I feel powerless in a psychotic crisis	18%	28%	54%
I feel unsafe near an acute psychotic patient	16%	35%	49%

**Need for continual professional development training (CPD training)**			

I need CPD training on guiding of and communicating with psychotic patients	55%	27%	18%
I need CPD training on interventions in a psychotic crisis	63%	20%	17%
I need CPD training on antipsychotic pharmacotherapy	54%	24%	22%

In the acute phase of psychosis, GPs viewed themselves as the first contact. They also felt responsible for the long term care in the chronic stage: monitoring somatic co-morbidity and taking care of repeat prescriptions. Regarding periodic physical checks, 80% of GPs considered this their task: 55% annually, 34% once half-yearly. Mental functioning should be monitored by a community psychiatric nurse and periodically checked by a psychiatrist belonging to the mental health service. The GPs felt it their task to support the family and provide information on the patient's condition.

Not all of the respondents felt capable of making contact with the patient during a psychotic crisis, whereas they were confident making contact with the family. Nevertheless, most GPs felt reasonably well-able to intervene in a crisis situation. A minority felt powerless or unsafe. GPs did indicate they needed further training in this area.

### Practice experiences

A majority of respondents (59%) had seen a patient concerning psychosis in the previous 6 months, and 78% had seen one in the last 12 months. These contacts included: first episode psychosis (21%), psychotic depression (23%), schizophrenia (29%), bipolar disorder (18%), psychosis resulting from drug abuse (3%) and the remaining category were: second episode of psychosis, organic psychosis and delirium.

Of these patients, 62% had been in contact with mental health services previously. Most cases (84%) were referred to a mental health service. A minority (39%) of GPs prescribed an antipsychotic drug themselves, of which 53% concerned a restart of previously prescribed drugs. Most GPs (60%) made follow-up appointments and contacted the family regularly (68%). At times, the GP would do a physical check (35%) or request lab checks (28%).

### Experiences with specialised mental health services (table 2)

**Table 2:** Experiences with specialised mental health services (N = 186)

	Agree	Neutral	Disagree
It does take a lot of effort to consult a psychiatrist in an emergency psychotic crisis	30%	9%	61%
My information is taken seriously by the psychiatrist	60%	25%	15%
The mental health services involve me, as GP, in the treatment plan	12%	20%	68%
The mental health services keep me informed on a regular basis	19%	29%	52%
I feel supported by the collaboration with the mental health services	38%	39%	23%

In emergency cases, 61% of GPs stated they were able to reach mental health care services easily, and they felt taken seriously as the referring party. However, there was no collaboration with mental health care providers in the organisation of long term care. GPs were not included in the development of treatment plans, and did not receive regular information on the patient's status during treatment.

#### Multivariate analysis

A factor analysis of the answers to the questions of the section 'Opinion and Task Perspective', the questions on collaboration with MHS, and the questions on personal details with regard to attitude and need for training identified the following four independent factors:

- self-experienced competencies in the acute phase of psychotic illness

- task perspective in the chronic phase of psychotic illness

- experience with mental health services

- need for continual professional development training (CPD)

### Relationship between the four domains and GP (practice) characteristics (table 3) and treatment aspects of GPs (table 4)

**Table 3:** Significance of the relationship between the four domains and GP (practice) characteristics

Domains	1	2	3	4
gender GP	.082	.355	.573	.624
type of practice	.704	.999	.704	.124
part time – fulltime	.397	.988	.247	.335
GP trainer	.493	**.014**	.488	.723
urbanization	.109	.059	.406	.767
work experience in psychiatry	**.002**	.266	.945	**.045**
work experience as GP	.066	.724	.936	.790
volume of practice list	.091	**.044**	.777	.102
registration of SMI in electronic patient records	.345	.121	.548	.806
estimated number of patients susceptible to psychosis	.111	**.003**	.996	.890

*statistically significant relations (p ≤ 0.05) are printed in bold

1 = self-experienced competencies in the acute phase

2 = task perspective in the chronic phase

3 = experience with mental health services

4 = need for CPD training

**Table 4:** Significance of the relationship between the four domains and GP's treatment aspects

Domains	1	2	3	4
most recent treatment concerning psychosis	**.021**	.748	.625	**.026**
referral patient to mental health services	.661	.206	.270	.658
start pharmacotherapy	.446	.243	.187	.771
follow-up appointment with patient	.184	**.013**	.642	.758
follow-up appointment with family	.279	.093	.977	.341
physical check	**.032**	.319	.332	**.050**
lab diagnostics	.093	.940	.466	.231

*statistically significant relations (p ≤ 0.05) are printed in bold

1 = self-experienced competencies in the acute phase

2 = task perspective in the chronic phase

3 = experience with mental health services

4 = need for CPD training

GPs who felt competent in the acute phase of a patient's psychotic illness suffered less from feelings of helplessness or fear than GPs who were not familiar with problems of patients with SMI. More often than not, these GPs had gained work experience in a psychiatric institute and felt less need for extra training. They had diagnosed a patient with acute psychosis more often in the last six months, and conducted physical checks more often. Also, they made follow-up appointments with patients more often, and considered periodic checks on a patient's self-neglect a part of the GP's responsibility.

GPs with a broader role perspective on the care for psychotic patients often had a smaller practice list and were more often GP trainers. They estimated that they had a higher prevalence of psychotic patients in their practice, which was consistent with actual figures: 1% of the population is susceptible to psychosis. A broader role perspective is associated with a higher job satisfaction in the guidance of psychiatric patients. This group of GPs made use of the community psychiatric nurse more often, if available.

No significant relation was found between practice characteristics and experience with mental health services.

## Discussion

The GPs responding to the questionnaire agree on their role in the acute phase: having assessed the patient's condition, they refer the patient to a mental health centre and/or prescribe an antipsychotic drug. In addition, the GPs support the family members. GPs consider the collaboration with mental health services as adequate in this phase. Not surprisingly, inexperienced GPs feel the need for training in dealing with crisis situations.

In the chronic phase, the responding GPs differ in their opinion as to what care they should provide. These differences are explained by different task perspectives, experience with regional mental health services, and their perceived need for specific training.

Many GPs do feel involved in this stage, but they find the psychosocial problems associated with it quite difficult. Currently, GPs tend just to diagnose and treat somatic co-morbidity, but a majority would be willing to monitor physical health in the future. They also want to be responsible for repeat prescriptions, but they lack expertise in the effectiveness and side effects of antipsychotic drugs.

GPs feel reluctant to inquire about the patient's self-care, and therefore they are unable to assess the risk of neglect. Possibly they are not aware of the fact that a better physical condition improves the psychiatric symptoms and the quality of life [5].

GPs that have a broad role perspective tend to monitor the patient in the chronic phase and experience a higher job satisfaction in the guidance of their patients than those who do not. As these GPs often have a smaller practice list, it is assumed that this enables the GP to know and understand his patients better.

The collaboration between GPs and mental health specialists in chronic cases leaves much to be desired. There is no cohesion in the care given. This problem appears to be universal [4,41,42]. The GP is not included in the development of treatment plans and is not informed about the patient's status during treatment. This finding was not associated with specific practice characteristics.

### Strengths and weaknesses

Although the response rate to the questionnaire was low, the responding GPs were comparable with the total group of Dutch GPs in gender, age, type of practice and location. This low response rate may have been due to different causes. First, the questionnaire comprised several items on practice routines in relation to the last patient seen. This type of question put a demand on the GP's memory and might take some time in retrieving the necessary details. Secondly, it is feared that GPs who lacked affinity with severe mental illnesses, simply did not answer the questionnaire. Nevertheless, the range of responses was quite wide. Perhaps the responders have sketched too positive image of primary health task perspectives. The findings however offer concrete possibilities to improve the actual care for patients with severe mental illness.

## Conclusion

The responding GPs find themselves capable of providing adequate care in the acute phase. As crisis situations are relatively rare, most of them feel a need for continual professional development training.

In the chronic phase of a psychotic illness, GPs are willing to be part of the care system surrounding a psychotic patient. Most GPs consider assessing the patient's physical condition and detecting and monitoring somatic co-morbidity as their responsibility. Risk management and the treatment of somatic co-morbidity are part of the GP's expertise, as is giving support and information to the patient's family. However, such judgment requires an active, outreaching attitude on the GP's part. When communicating with chronic psychotic patients, it is necessary for GPs to take the patient's possible cognitive handicaps into account [43].

The majority of the GPs, however, experience the need for training in counselling in the chronic phase, specifically in pharmacotherapy, including topics like side-effect and interactions.

The collaboration with mental health services is less than optimal and should be improved. With regard to the patients' perspective [44], the concept of continuity of care refers also to the firm inclusion of the GP within comprehensive multidisciplinary care. The GP deserves a central position especially with respect to somatic co-morbidity and (psycho)pharmacological interactions.

Psychiatrists, like most specialists [45], consider "referring" to be the GP's primary task. They do not consider GPs as co-consultants in the care system surrounding a patient with SMI. GPs may be able to change this, through focusing more on the health condition of chronic psychiatric patients, and describing their tasks in a set of guidelines [46].

## Recommendations

It is recommended that the responsibilities and tasks for GPs dealing with severe mental illness should be developed within multidisciplinary guidelines. These guidelines should be consistent with GPs' competences, especially those of monitoring and treating somatic co-morbidity, giving support and information to the patient's family, crisis intervention and prescriptions.

It is felt that a coherent care system should be created with the role of the GP clearly defined in the chain of care and, as a result, this care system would contribute greatly to the quality of care for patients with SMI.

## Competing interests

The authors declare that they have no competing interests.

## Authors' contributions

MO initiated the study and reviewed the literature. JS and MO supervised a student in writing the first draft of the questionnaire, and conducting a pilot study. CS obtained funding for the study. All authors contributed to the development of the questionnaire. MO carried out the data collection. KG performed the multivariate analysis. MO drafted the manuscript and the tables. All authors commented substantially on subsequent versions of the manuscript and all authors have approved the final version.

## Pre-publication history

The pre-publication history for this paper can be accessed here:


